# The effect of surface area on the properties of shape-stabilized phase change material prepared using palm kernel shell activated carbon

**DOI:** 10.1038/s41598-020-72019-1

**Published:** 2020-09-14

**Authors:** Ahmad Fariz Nicholas, Mohd Zobir Hussein, Zulkarnain Zainal, Tumirah Khadiran

**Affiliations:** 1 Materials Synthesis and Characterization Laboratory, Institute of Advanced Technology (ITMA), Universiti Putra Malaysia, 43400 Serdang, Selangor Malaysia; 2 Forest Product Division, Forest Research Institute Malaysia (FRIM), 52109 Kepong, Selangor Malaysia

**Keywords:** Materials science, Nanoscience and technology, Physics

## Abstract

The effect of the surface area of palm kernel shell activated carbon (PKSAC) on the properties of n-octadecane-encapsulated shape stabilized phase change material (SSPCM) for thermal energy storage (TES) application were studied. Various surface areas of the PKSAC were prepared using different amounts of H_3_PO_4_ treatment given to palm kernel shells from 0, 5, 10, 30 and 40% before the activation. The impregnation of n-octadecane into the different surface areas of PKSACs produced SSPCMs with different physico-chemical characteristics. The DSC analysis indicates that the higher the surface area of the PKSAC resulted in the higher freezing temperature due to the higher PCM loading that was encapsulated into the PKSAC pores. The results obtained from XRD, FESEM, Raman spectroscopy, TGA/DTG and leakage study indicate that the PKSAC is a good framework material for the development of n-octadecane-encapsulated SSPCM. It was also found that the surface area and porosity of the frameworks, activated carbon play an important role on the PCM loading percentage and their ability to be used as a thermal energy storage material.

## Introduction

Thermal energy storage (TES) for buildings has gained much attention recently. Many different techniques of TES preparation were developed over the past decades such as underground thermal energy storage, building thermal mass utilization, energy storage tanks and phase change materials (PCM)^1^. The energy storage system is commonly available in various types but it application is usually determine by cost, low density, low volume of storage and limited efficiency^2^. Manufacturing, commercial, industrial and residential sectors' demands were high towards the TES to reduce their cost of production. These demands lead to the development of TES systems that match specific applications^3^. The demands are more towards advantages such as cost savings, efficiency in the utilization of raw materials and the efficiency that can be achieved using the chosen TES.

The increase of the internal building temperature caused by sunny days can be reduced and maintained, while cold, windy or rainy nights can be heated by the release of the energy that stored earlier by TES. The applications of TES in the buildings benefit the users by reducing the heating and cooling cost, the size costs of components and improved indoor environmental quality^1^. Furthermore, the pollution caused by conventional energy sources used can be reduced by the enhancement of renewable energy applications in different sectors^4^.

PCM is commonly used as heat storage due to its special characteristics such as having a wide range of melting and solidifying temperature and high storage density^5^. PCM is latent heat storage (LHS) which able to change its phase based on its melting and freezing temperature. As compared to sensible heat storage (SHS) such as water, sand, molten salts and rocks, the storage density of PCM is more than five times higher^6^. Even though the SHS is much cheaper, its energy density is lower, which requires a high volume in its application. It is generally considered that LHS has a better storage capability compared to SHS.

LHS also has problems during heat storage in which it experiences density changes, low thermal conductivity, short life spends, caused corrosion, phase segregation and sub-cooling^3^. There are several approaches to solve these problems and one of them is by the impregnation of PCM (e.g. n-octadecane) into a framework material that is used for solid–liquid PCM. Several high conductivity additive materials such as aluminum powder, graphite and metal foams were used to enhance the performance of solid–liquid PCM^7^. In order to enhance the thermal conductivity of PCM, the aluminum powder was added to the PCM to ameliorate the PCM and increased the distiller productivity of the single slope solar water distiller^8^.

The addition of 10% expended graphite (EG) to PCM for the thermal conductivity for latent heat thermal energy storage (LHTES) applications were found promising for the LHTES application^9^. In addition, PCM embedded with metal foam could enhance its heat transfer ability, where the metal foam could enhance the heat transfer rate during the melting process by natural convection. The solidification process test shows that the application of metal foams can trigger the sample solidified much faster than pure PCM. The result is parallel with the two-dimensional analysis which proves that the use of metal foams substantially increases the heat transfer performance of the PCM^10^. Recent study by Sari et al. on the preparation and evaluation of shape-stabilization dodecyl alcohol (DDA) shows that the activated charcoal prepared from waste tire was able to prevent the leakage of melted DDA during the phase change process^11^.

Polymers such as high-density polyethylene, poly-(ethylene oxide) and ethylene propylene diene terpolymer plastic also can be used as supporting materials. The use of polyethylene glycol (PEG) as the PCM and its incorporation with palygorskite (Pal) clay in the preparation of form-stable composite PCM (F-SCPCM) has been studied. It has shown a promising option for the formation of thermal energy storage^12^, eventhough some polymers cause health and environmental issues. Due to these reasons, researchers are preferred to use inorganic porous materials such as carbon nanotubes, graphene oxide, expanded graphite, SiO_2_ composites, expanded perlite, activated montmorillonite and activated carbon as supporting materials^13^. Work by dispersing paraffin in high density polyethylene (HDPE) as the supporting material for the preparation of shape-stabilized paraffin to prevent the leakage of paraffin during melting and freezing processes was also done. It was found that the shape-stabilized paraffin is a new type of latent heat storage material which can maintain the shape in a solid state when the paraffin melts without encapsulation^14^.

Due to high surface area, the high volume of pores, high graphitic structure, chemically stable, abundant and relatively cheap, activated carbon has received much attention in various fields of science and technology^15^. Activated carbon can be produced from a variety of waste materials especially the agricultural industry, and this is actually good to reduce the waste biomass from the environment and turn it into useful products. Activated carbon can be used as the frameworks for PCM has the potential to shield the PCM from the external surrounding, increase the heat tranfer, control the evaporation rate and increase the thermal conductivity during the application and operation^16^. The physico-chemical properties of activated carbon like the surface area, pore width and surface chemistry are vey much depending on their method of preparation and the raw material used. Therefore, the physico-chemical properties of the activated carbon derived from palm oil based wastes will not give similar pore size distribution, geometrical shape, network inner connection, and functional groups on their pore surface^17^.

In this study, the encapsulation of PCM into the pores of activated carbon was accomplished, to produce shape-stabilized phase change materials (SSPCM) and to be subsequently used for thermal energy storage (TES) application. In the preparation of SSPCM, palm kernel shell (PKS) was valorized as a bio-source for the production of the activated carbon and n-octadecane was used as the heat storage medium. In addition, this study is conducted to investigate the effect of the surface area of PKSAC as the starting material towards the properties of the resulting SSPCM prepared from them.

## Materials and mathod

### Materials

The palm kernel shell (PKS) was collected from Seri Ulu Langat Palm Oil Mill, Dengkil Selangor. Water was used to wash and clean the PKS samples before they were dried. The dried samples were then crushed into finer pieces using a stable arm grinder before they were treated with H_3_PO_4_. In the preparation of activated carbon, activating agent such as KOH, ZnCl_2_, K_2_CO_3_, and H_3_PO_4_ are usually used, but the most popular activating agents were ZnCl_2_ and H_3_PO_4_^18^. Activating agents like KOH and K_2_CO_3_ cause carbon gasification and the formation of hydrogen, which will not contribute to the increase in the carbon yield^19^. On the other hand ZnCl_2_ is less preferable compared to H_3_PO_4_, as it is less environmental friendly and more corrosive with inefficient chemical recovery^20^. After that, 20 g of the crushed PKS sample were weighed and treated with 100 mL of different concentrations (0, 5, 10, 20, 30 and 40%) of H_3_PO_4_, orthophosphoric acid (85%) (SystermChemAR, Malaysia). The treatment was done for 24 h under room temperature before the H_3_PO_4_ solution was filtered out. The filtered samples were then oven dried at 70 °C before the carbonization process.

### Carbonization

The PKS samples were weighed (5 g each) before they were carbonized using an electrical tubular furnace in the one step activation method. Each sample that was treated at different concentrations of H_3_PO_4_ (0, 5, 10, 30 and 40%) were carbonized at 500 °C for 2 h holding time and 10 °C min^−1^ heating rate. This was the optimum conditions for the preparation of activated carbon from PKS under our experimental conditions, where the highest specific surface area was obtained^21^. After carbonization, the palm kernel shell activated carbons (PKSAC) were crushed using a mortar before it was cleaned three to four times using boiled distilled water. The cleaned samples were oven dried to remove internal moisture. The samples were then weighed and recorded again before they were kept into vials and labeled as C0, C5, C10, C30 and C40 for treatment with 0, 5, 10, 30 and 40% of H_3_PO_4_, respectively and used for further treatment and analyses.

### Preparation of a shape-stabilized phase change material

The simple impregnation method was adopted for the production of a shape-stabilized phase change material (SSPCM). About 1.8 g of n-octadecane paraffin wax was weighed before it was melted at its melting temperature and disintegrated in 30 mL absolute ethanol, ethyl alcohol (99.7%) (R&M Chemicals, Malaysia). Then, 2 g of the PKSAC sample was mixed with the dissolved n-octadecane and stirred at 600 rpm for 4 h. The suspension was then oven-dried at 80 °C for 48 h or until all the surplus ethanol was evaporated. The final product of SSPCM was stored in a vial and labeled as SSPCM-C0, SSPCM-C5, SSPCM-C10, SSPCM-C30 and SSPCM-C40 for PKSAC obtained by treatment with 0, 5, 10, 30 and 40% of H_3_PO_4_, respectively, for further use and characterization.

### Sample characterization

The surface area and porosity of PKSACs (C0, C5, C10, C30 and C40) and their SSPCM nanocomposites (SSPCM-C0, SSPCM-C5, SSPCM-C10, SSPCM-C30 and SSPCM-C40) was measured by the Brunauer-Emmet-Teller (BET) nitrogen gas adsorption–desorption method. The analysis was done at 77 K using an accelerated surface area and porosimeter, Micromeratics Tristar II plus (Micromeratics, Norcross, GA, USA). This method was applied to identify if the n-octadecane has successfully encapsulated into the PKSAC or just covered the outer surface. Before the measurements, the samples were degassed at 290 °C for 9 h under vacuum and the BET and BJH (Barret-Joyner-Halenda) equations were used to determine the specific surface area and pore size distribution of the samples, respectively^21^.

The X-ray diffraction (XRD) patterns of PKSACs (C0, C5, C10, C20, C30 and C40), n-octadecane and SSPCMs (SSPCM-C0, SSPCM-C5, SSPCM-C10, SSPCM-C30 and SSPCM-C40) were obtained using a Shimadzu XRD-6000 XRD (Kyoto, Japan). The PKSACs were analyzed at room temperature while n-octadecane and SSPCM were analyzed at 27 ± 5 °C with a scanning range and rate of 10–35° (2θ) and 4° min^−1^, respectively for all the samples^17^.

TGA/DTG thermal analyses were obtained using a Q500 V20.13 Build 39 (TA Instruments, Lukens Drive, New Castle, DE, USA). It is used to identify the thermal stability of the PKSACs (C0, C5, C10, C30 and C40), n-octadecane and SSPCMs (SSPCM-C0, SSPCM-C5, SSPCM-C10, SSPCM-C30 and SSPCM-C40). The experiment was conducted using 10 mg of the samples and heated under the nitrogen atmosphere at 5 °C min^−1^ heating rate from room temperature to 1,000 °C^17^.

The chemical properties of the PKSACs (C0, C5, C10, C30 and C40), n-octadecane and their SSPCM nanocomposites (SSPCM-C0, SSPCM-C5, SSPCM-C10, SSPCM-C30 and SSPCM-C40) was done using the fourier-transform infrared spectroscopy (FTIR) on a Perkin Elmer BX FTIR spectrophotometer (Waltham, Massachusetts, USA) with the KBr method at room temperature. The FTIR spectra were recorded at 400–4,000 cm^−1^^17^.

The thermal energy storage properties, such as melting and freezing temperature besides enthalpy (latent heat) of the pure n-octadecane and SSPCM nanocomposite were analyzed by a differential scanning calorimeter (DSC), a Mettler Toledo 822e, together with a refrigerated cooling system. About 6 ± 1 mg of the samples were weighed into aluminum pans. For the heating cycle stage, the measurement was performed at − 20–70 °C and vice versa for the cooling cycle stage under a constant flow of nitrogen atmosphere with the flow rate of 60 mL min^−1^. The impregnation efficiency of n-octadecane in the composites was calculated using the following equation:

1 $$ {\text{PCM content in composite }}\left( {{\text{wt}}\% } \right)  =  \left( {\Delta {\text{H}}_{{\text{m}}} / \, \Delta {\text{H}}_{{{\text{PCM}}}} } \right) \, \times 100 $$

where ∆H_m_ is the enthalpy of melting for the analyzed SSPCM nanocomposite (J g^−1^) and ∆H_PCM_ is the enthalpy of melting for the pure n-octadecane (J g^−1^)^13^.

The surface morphology and microstructure of the PKSACs (C0, C5, C10, C30 and C40) and SSPCM nanocomposites (SSPCM-C0, SSPCM-C5, SSPCM-C10, SSPCM-C30 and SSPCM-C40) were obtained using a NOVA NANOSEM 230 FESEM (field emission scanning electron microscope). The dried samples were spread on a conductive carbon adhesive tape surface that was attached to a FESEM stub and then gold-coated prior to analysis^22^.

The leakage study was done by keeping the SSPCM nanocomposites (SSPCM-C0, SSPCM-C5, SSPCM-C10, SSPCM-C30 and SSPCM-C40) for 3 days inside an oven at 100 °C. Before this process, the nanocomposites were left at 30 °C for 8 h to see their capability to keep the n-octadecane during the melting phase. The weight of the nanocomposite before and after it was exposed to 100 °C for 3 days was recorded^13^.

## Results and discussion

### Surface area and porosity of PKSACs and their SSPCMs

Raman spectroscopic analysis shows that all the PKSACs contain high graphitic structure with different quantities which indicates that the sample is strong enough to be used as the frameworks material for PCM^21^. The preparation of SSPCMs was done for all the activated carbons prepared in this work; C0, C5, C10, C30 and C40 by the impregnation of n-octadecane, the PCM into the pores of PKSACs, the host frameworks. This is to see the effect of the surface area on the physico-chemical properties of the resulting SSPCMs.

**Figure 1:**
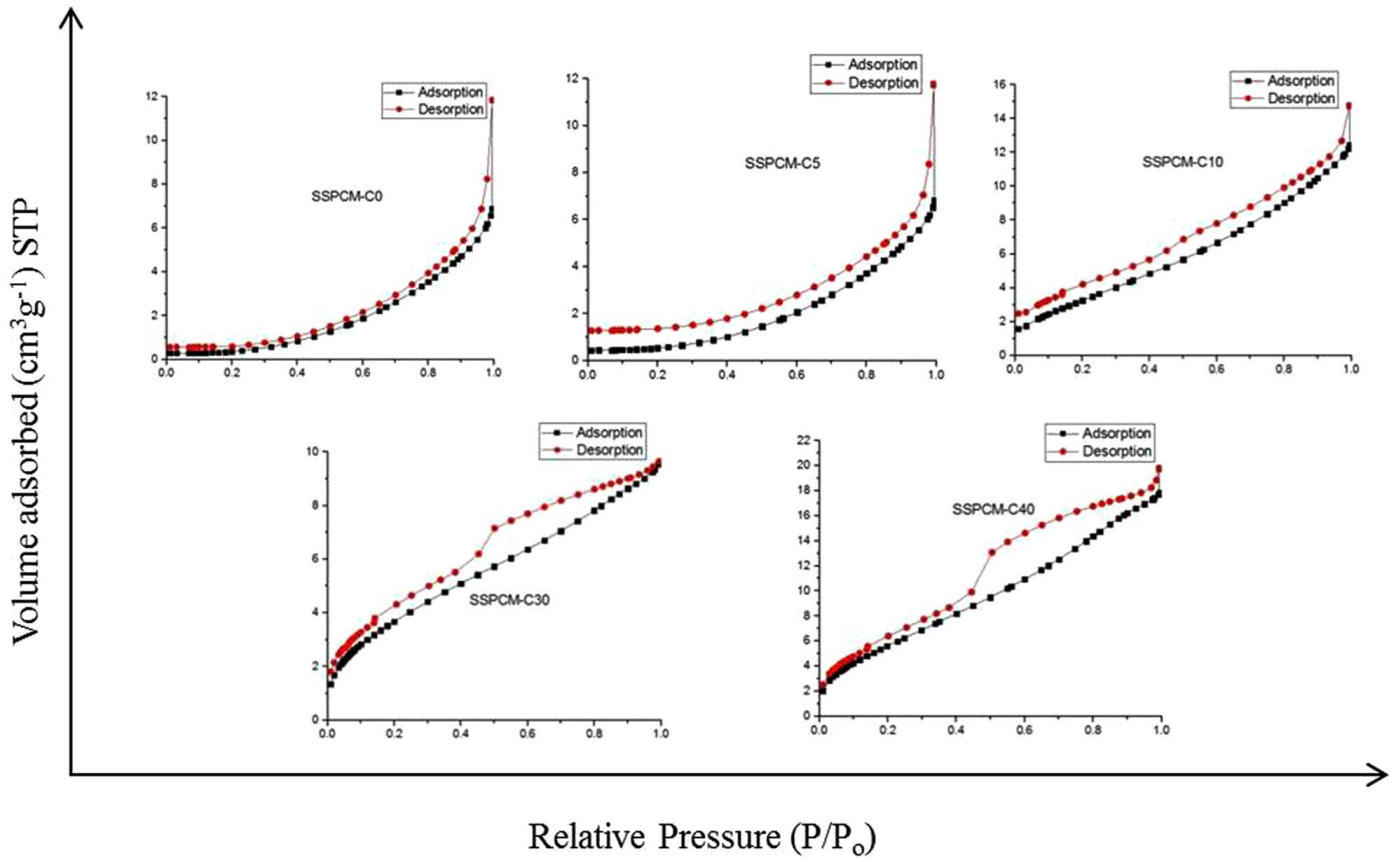
Nitrogen gas adsorption–desorption isotherm of SSPCMs.

Figure 1 shows the adsorption–desorption isotherms of SSPCMs prepared using C0–C40 after they were impregnated with n-octadecane_._ The isotherms for PKSACs are dominated by Type I and this changed to Type III for SSPCM-C0, SSPCM-C5 and SSPCM-C10, and Type IV for SSPCM-C30 and SSPCM-C40. That is indicating that the resulting SSPCM nanocomposites are of nonporous material. This is because the pores of the host, PKSACs were fully occupied by the PCM. The changes from Type I in the PKSAC to Type III and IV for the SSPCMs indicate that n-octadecane was successfully impregnated into the pores of PKSACs.

### X-ray diffraction

**Figure 2:**
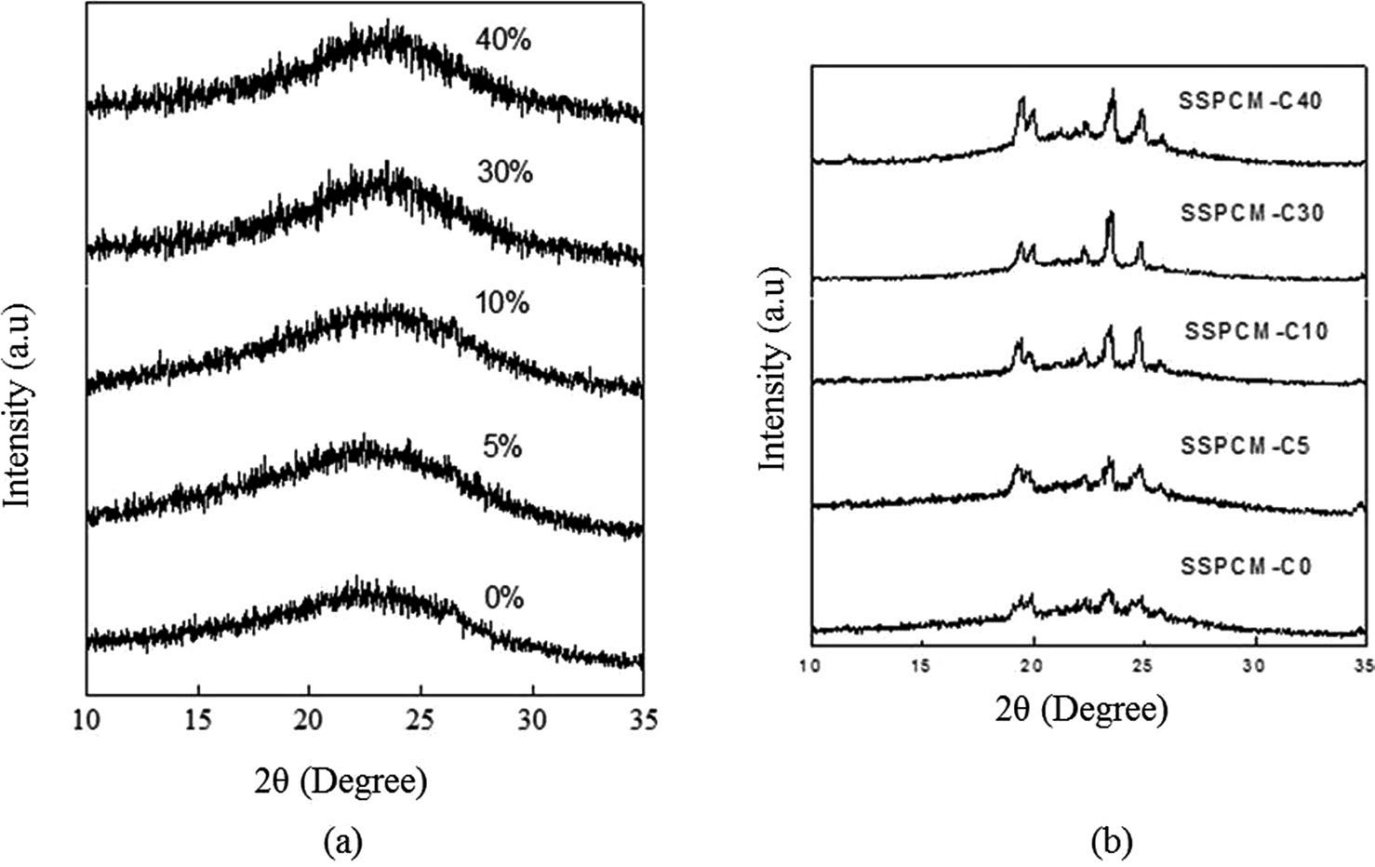
X-Ray diffraction patterns of (**a**) PKSACs and (**b**) their SSPCMs.

Figure 2a,b show the XRD patterns of PKSACs and SSPCMs, respectively. The PKSACs shows non crystalline structure which is the typical XRD pattern for activated carbon due to their amorphous character. In contrast, the pure n-octadecane displayed a crystalline pattern with peaks located at 2θ = 19°, 23° and 25°. The SSPCMs show a similar XRD pattern to the pure n-octadecane together the the hump, indicating the existence of n-octadecane in the AC framework. It also indicates that a similar peak exists in each of the SSPCM. This result indicates that all the ACs that were prepared in this work have the ability to hold the PCM, n-octadecane.

### Thermal properties

**Figure 3:**
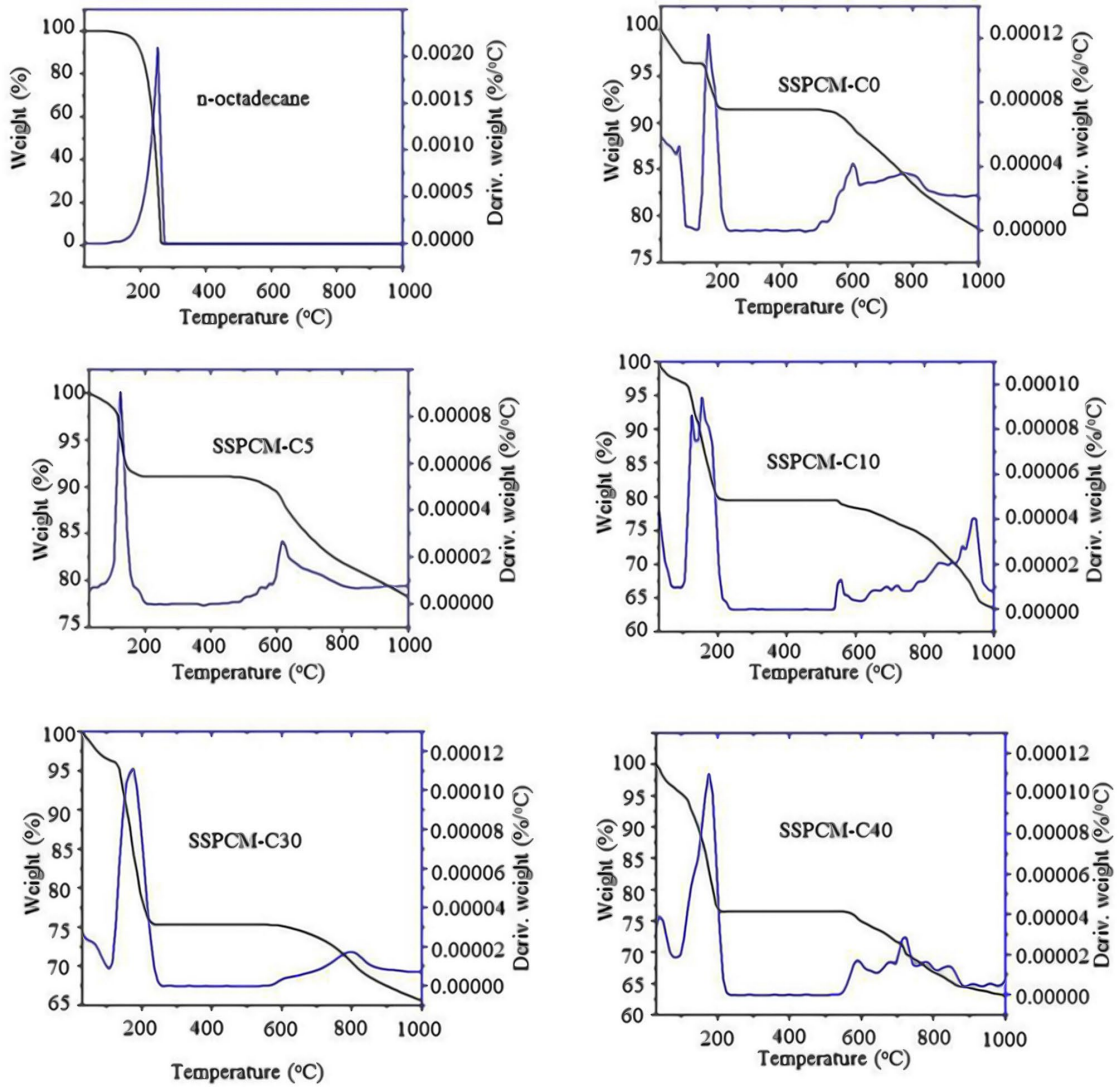
TGA/DTG thermograms of pure n-octadecane and SSPCM-C0, SSPCM-C5, SSPCM-C10, SSPCM-C30 and SSPCM-C40.

It is important to ensure that the material used for TES is thermally stable. The TGA/DTG techniques are used to check the thermal stability of the SSPCM nanocomposites. Figure 3 shows the TGA/DTG thermograms of pure n-octadecane and SSPCM (C0, C5, C10, C20, C30 and C40). The result shows minimal weight loss, indicating that there is evaporation of water due to the moisture adsorbed on the nanocomposite which is typically observed for all of the ACs prepared in this work. In addition, all of the ACs prepared, show thermal decomposition at a temperature above 500 °C, which is due to the pyrolysis that has taken place only until 500 °C (Supplementary Data). The thermal decomposition also occured to all the SSPCMs as shown in Table 1. Further pyrolysis occurred when the sample was exposed to a temperature above 500 °C. This result indicated that the AC prepared is thermally stable until 500 °C.

**Table 1:** Thermal properties of ACs, pure n-octadecane and their SSPCMs.

Sample name	T_1_–T_2_	T_m_ (°C)	Δ_m_ (mg)	Weight loss (%)	Total weight loss (%)
n-octadecane	94–273	261	10.69	100	100
C-0	40–114	75	0.48	4.48^a^	20.30
	547–839	790	1.68	15.82^c^	
C-5	37–134	75	0.62	6.00^a^	22.22
	518–882	792	1.68	16.22^c^	
C-10	46–104	65	0.55	5.28^a^	19.01
	499–927	878	1.43	13.73^c^	
C-30	56–114	94	0.73	6.95^a^	24.87
	508–878	789	1.89	17.92^c^	
C-40	55–94	65	0.46	4.38^a^	20.58
	528–917	819	1.74	16.20^c^	
SSPCM-C0	42–107	97	0.37	3.60^a^	21.28
	104–214	151	0.50	4.93^b^	
	509–859	772	1.30	12.75^c^	
SSPCM-C5	47–75	65	0.42	3.86^a^	21.09
	105–185	148	0.55	5.03^b^	
	528–820	607	1.33	12.20^c^	
SSPCM-C10	31–116	65	0.47	4.35^a^	36.60
	126–205	178	1.74	16.17^b^	
	550–882	723	1.73	16.08^c^	
SSPCM-C30	40–115	59	0.38	3.48^a^	33.50
	125–220	204	2.30	21.20^b^	
	558–919	794	0.96	8.82^c^	
SSPCM-C40	45–119	80	0.54	5.16^a^	36.90
	130–213	173	1.93	18.33^b^	
	530–861	790	1.41	13.41^c^	

Decomposition of water^a^, n-octadecane^b^ and activated carbon^c^.

As expected, the TGA/DTG thermograms show 100% weight loss for pure n-octadecane was observed at 94–273 °C, which indicates that the n-octadecane is relatively pure. Generally, the TGA/DTG thermograms for SSPCM-C0, SSPCM-C5, SSPCM-C10, SSPCM-C30 and SSPCM-C40 illustrate three phases of decomposition. The evaporation of water occurred at the first stage and followed by the decomposition of n-octadecane and further pyrolysis of AC. Table 1 shows the TGA/DTG thermograms of C20, pure n-octadecane and SSPCMs (C0, C5, C10, C30 and C40). The result indicates that decomposition occurred for all the SSPCM samples but at different weight loss of n-octadecane. This indicates that different loading percentages of the n-octadecane into the pores of different ACs with the different surface areas. SSPCM-C30 shows the highest weight loss percentage of n-octadecane which is 21.20% followed by SSPCM-C40 (18.33%), SSPCM-C10 (16.17%), SSPCM-C5 (5.03%) and SSPCM-C0 (4.93%). These TGA/DTG thermograms analyses show that SSPCM-C30 has the highest PCM loading percentage which indicates that the C30 framework can be stored and hold more PCM compared to the other PKSACs due to its highest surface area.

**Figure 4:**
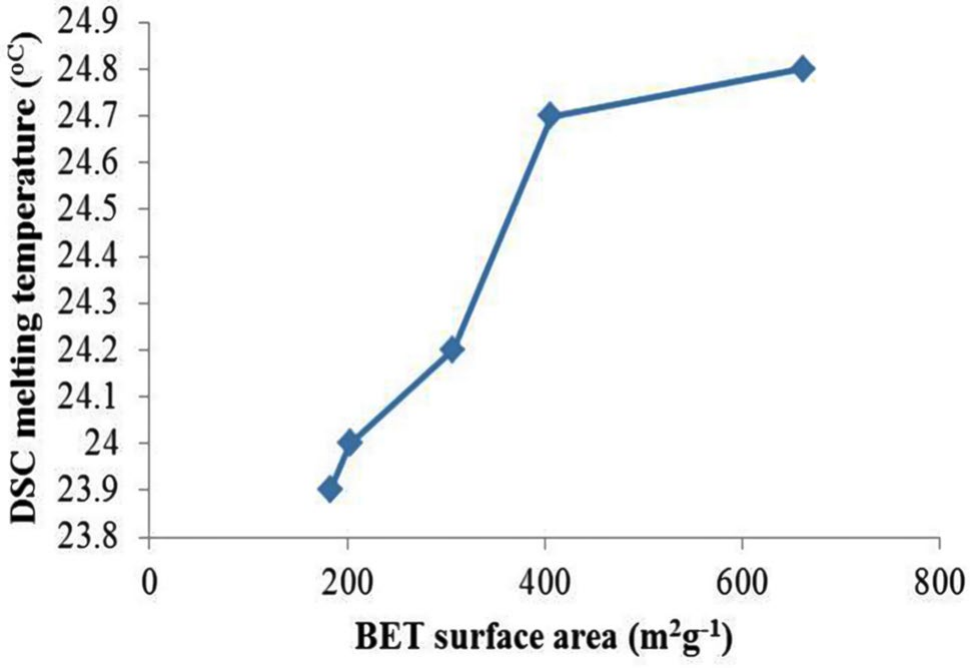
Plot of BET surface area (m^2^ g^−1^) against PCM weight loss (%) obtained by the TGA/DTG analysis for different BET surface areas of activated carbons used as the host materials.

Figure 4 shows a plot of BET surface area versus weight loss of PCM prepared for various PKSACs. The high surface area of PKSAC used to produce SSPCM resulted in a high weight loss of PCM due to more PCM that can be encapsulated into the pores of PKSAC. This linear correlation between the surface area of the PKSAC and PCM loading percentage indicates that the surface area of the PKSAC will directly effect the amount of PCM loading percentage in the production of SSPCM. The higher the surface area of the PKSAC, more PCM can be loaded into the pores, therefore the higher the PCM weight loss was observed by TGA/DTG analyses.

### Fourier-transforms infrared spectroscopy

**Figure 5:**
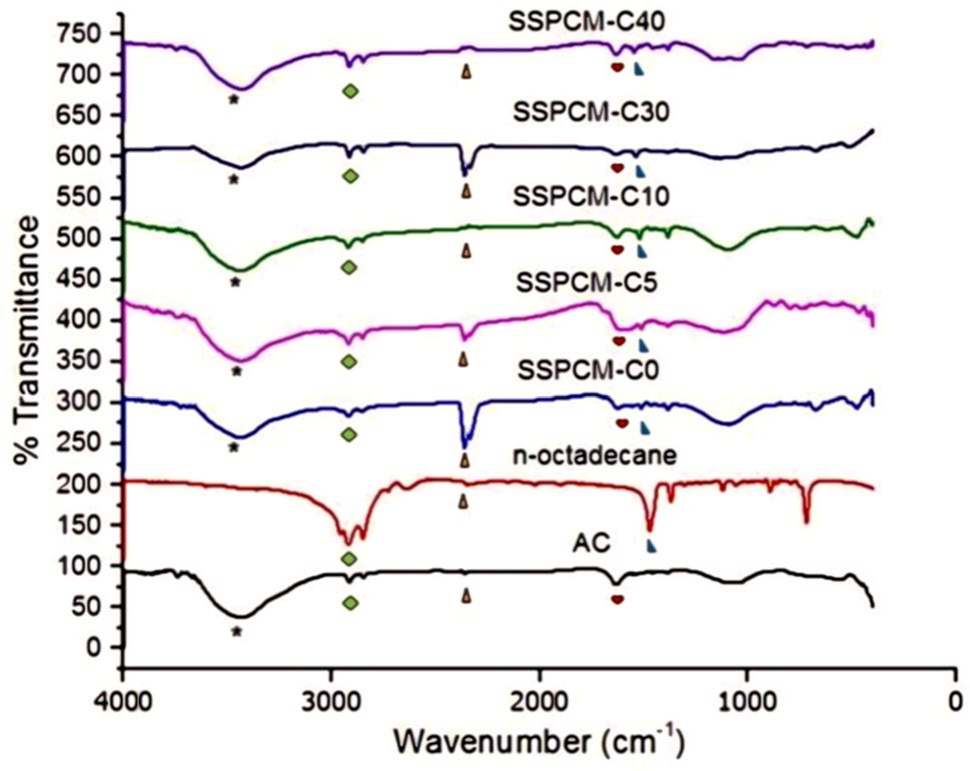
FTIR spectra of AC, n-octadecane, SSPCM-C0, SSPCM-C5, SSPCM-C10, SSPCM-C30 and SSPCM-C40 (The symbols on FTIR spectra are assigned to FTIR adsorption peak; (filled black star) 3,430 cm^−1^, (filled green rhombus) 2,919 cm^−1^, (filled orange triangle) 2,360 cm^−1^, (filled red heart) 1,635 cm^−1^, (filled blue right triangle) 1,470 cm^−1^).

Chemical characterization of n-octadecane and SSPCMs (C0, C5, C10, C30 and C40) was determined using FTIR spectroscopy as illustrated in Fig. 5. The main absorption band of AC is observed in the region of 3,650–2,500 cm^−1^ due to O–H stretching of the adsorbed moisture. The existence of an absorption band at 2,360 cm^−1^ is due to the CO_2_ band from the sample and also from the external environment. The bands at 1,635 and 1,537 cm^−1^ matched to the aromatic ring carbon of C=C stretching of the lignin^23^. An absorption band at 1,385 cm^−1^ is due to the CH_2_ bending vibration^24^.

The FTIR spectra of n-octadecane shows the C–H stretching band which corresponds to the major absorption band for the n-octadecane in the region of 2,975–2,850 cm^−1^. Due to the CH_2_ bending vibration in n-octadecane, the absorption band shows at 1,470 cm^−1^^25^. There is the integration of O–H and C–H stretching modes on the FTIR spectra of the SSPCM nanocomposites corresponds to the absorption band in AC and n-octadecane. No chemical interaction between the n-octadecane and AC can be clearly observed. The possible interaction is due to the capillary and surface tension forces (cohesion and adhesion) between the AC pore walls and the paraffin. That is the advantage as the forces is needed to prevent the liquid leakage during the phase conversion process.

### Surface morphology

**Figure 6:**
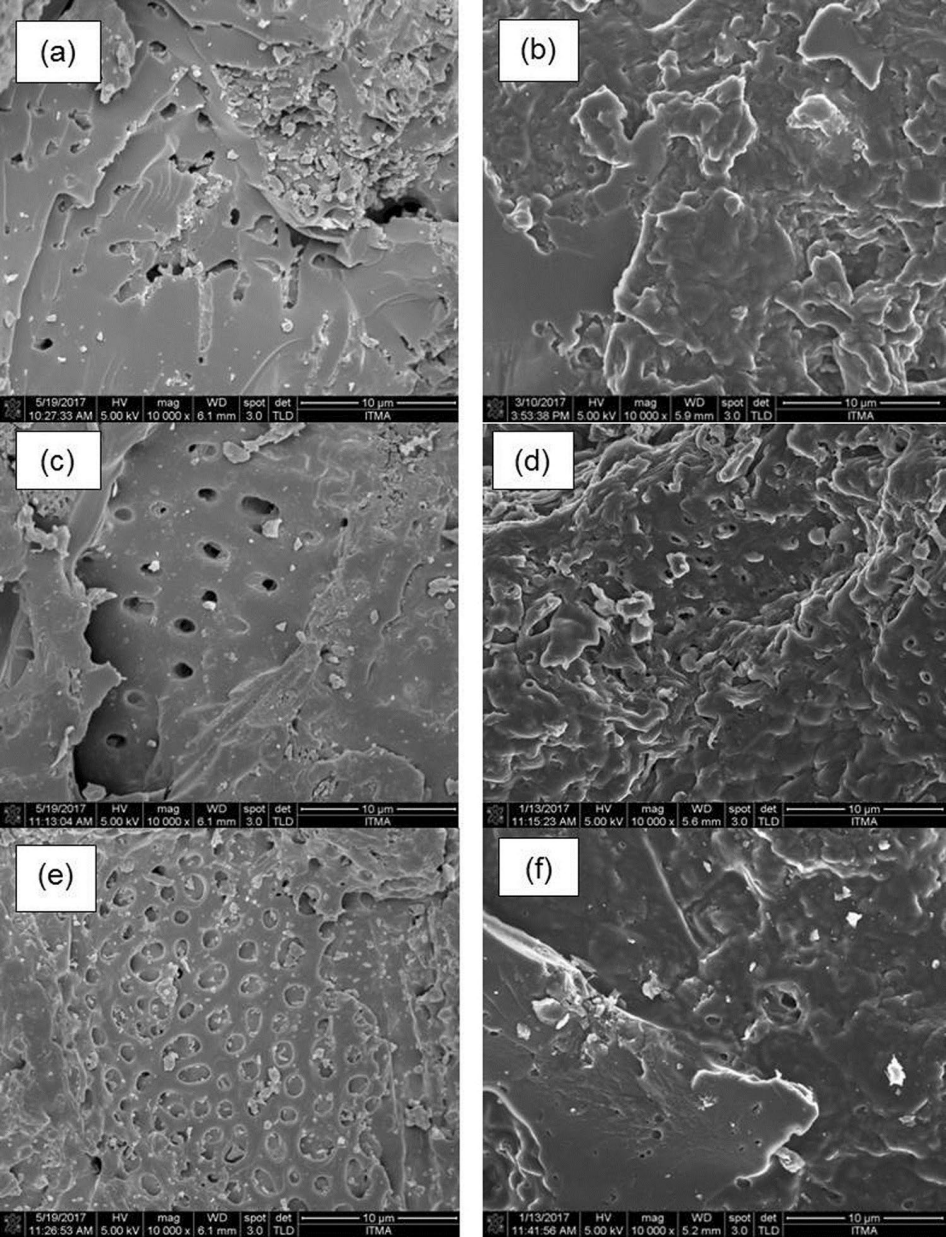
Field emission scanning electron microscopy images of (**a**) C5 (**b**) SSPCM-C5 (**c**) C30 (**d**) SSPCM-C30 (**e**) C40 (**f**) SSPCM-C40, viewed under ×10,000 magnifications.

The surface morphology of the nanocomposites was observed by FESEM, which is crucial to observe the transformation of the physical structure before and after the PCM was impregnated into the AC pores. Figure 6 shows the FESEM images of some typical ACs and their SSPCMs; C5, SSPCM-C5, C30, SSPCM-C30, C40 and SSPCM-C40. Figure 6a,c,e show the images of PKSACs, having a well-developed porous structure, various pore sizes of irregular in shape. Figure 6b,d,f, on the other hand, show that the n-octadecane was completely absorbed into the pores network of PKSACs and dispersed homogeneously, thus indicating that the pores were covered by n-octadecane due to the encapsulation of the n-octadecane.

### Effect of surface area of PKSAC on the on TES properties of the SSPCM

**Figure 7:**
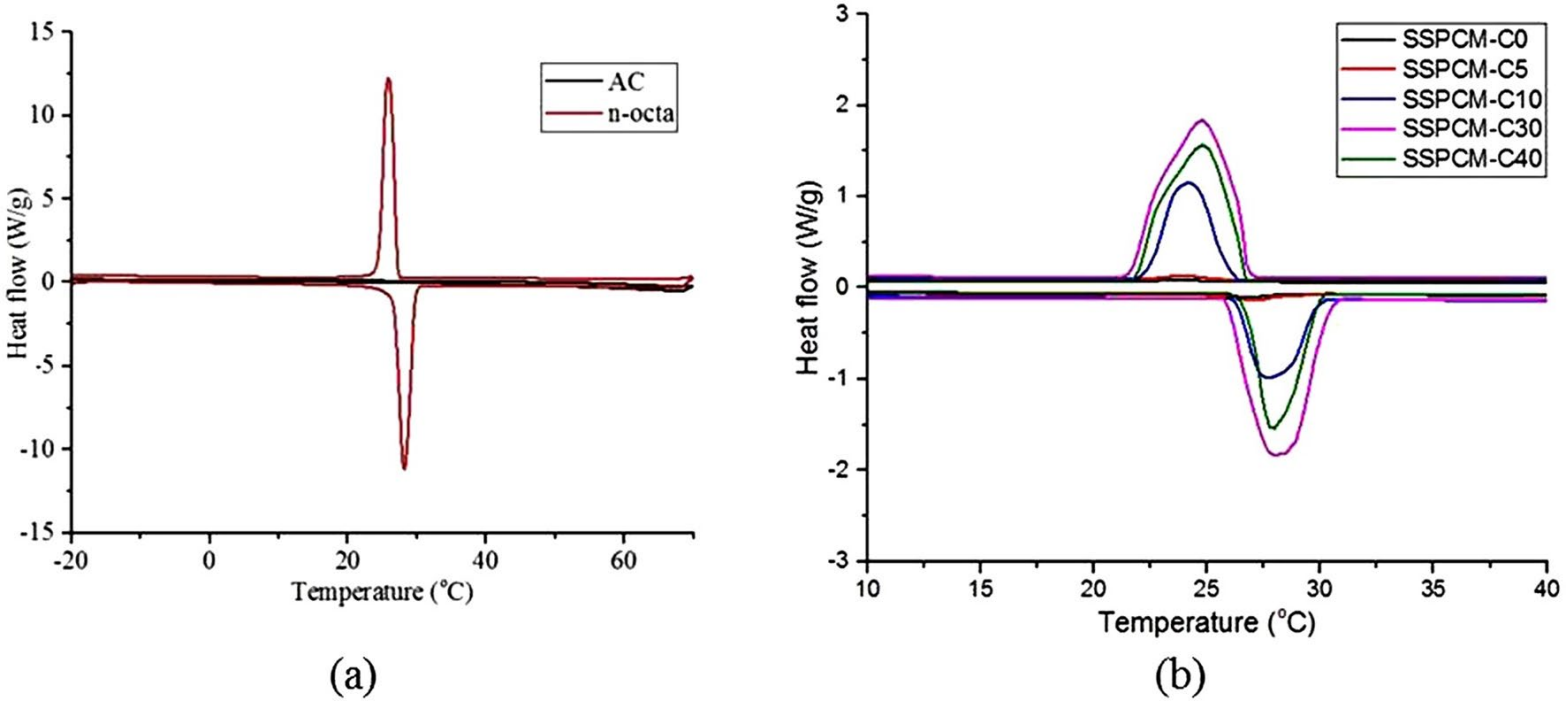
Differential scanning calorimeter thermograms of (**a**) ACs and pure n-octadecane, and (**b**) SSPCM-C0, SSPCM-C5, SSPCM-C10, SSPCM-C30 and SSPCM-C40.

The phase conversion analysis of the SSPCM nanocomposites was studied using the DSC method. The analysis was done to determine the melting and freezing activity of the SSPCM nanocomposites and the chemical interaction between the n-octadecane and the AC. Figure 7a shows the solid–liquid melting and liquid–solid freezing peaks of AC and pure n-octadecane. As expected, there is no activity for AC due to the non-appearance of n-octadecane. Activated carbon shows the absence of solid–liquid melting and liquid–solid freezing peak which is the typical pattern for pure activated carbon. Pure n-octadecane shows the peak of melting and freezing which indicates that the heat absorbed and released activity happened.

DSC peaks of SSPCM nanocomposites in Fig. 7b show that there is melting and freezing activity for the samples, indicating that the n-octadecane was successfully impregnated into the pores of the ACs. The slight difference in the intensity of heat flow indicates that the SSPCM-C30 is the highest followed by SSPCM-40, SSPCM-C10, SSPCM-C5 and SSPCM-C0. This intensity indicates that the SSPCM-C30 has the highest thermal energy storage capacity compared to the other SSPCMs.

The pure n-octadecane and SSPCMs samples show similar thermal properties compared to their corresponding ACs, indicating that the n-octadecane was not reacted with AC. However, the SSPCM shows lower melting and freezing temperature ranges compared to pure n-octadecane as the n-octadecane in the SSPCM was encapsulated into the pores of ACs. In addition, SSPCM-C30 showed a higher temperature range compared to other SSPCMs which shows that the C30 is able to store and hold more n-octadecane compared to other ACs. The high surface area of C30 resulted in a higher amount of n-octadecane that can be stored in the pores, therefore increased the molecular motion of n-octadecane during the phase change process. It is also shown that SSPCM-C30 can store and release more thermal energy compared to the other SSPCMs. This is due to the higher loading of PCM.

**Figure 8:**
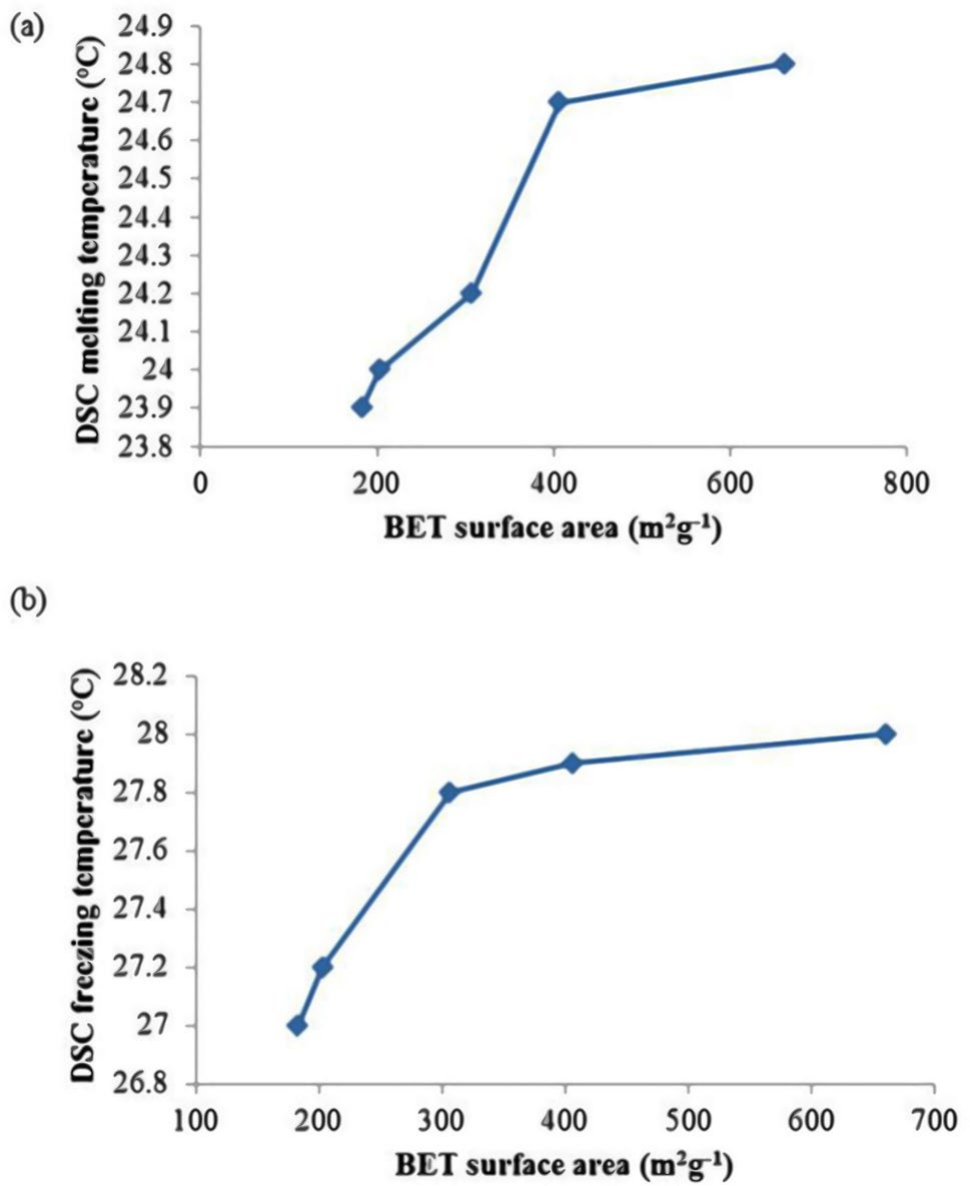
Plot of (**a**) DSC melting temperature (°C) and (**b**) freezing temperature (°C) against the BET surface area (m^2^ g^−1^) of the PKSACs.

Figure 8a,b shows a plot of DSC melting and freezing temperature (°C) of SSPCMs against the BET surface area (m^2^ g^−1^) of their corresponding PKSACs. This indicates the higher the surface area of the PKSAC resulted in higher freezing temperature due to higher PCM that was encapsulated into the PKSAC pores.

Table 2 summarizes the surface area/porosity and latent heat of the SSPCM nanocomposites before and after the leakage study. The latent heat of all the SSPCMs before and after the leakage study shows no significant difference. This is because the wall of the pores and n-octadecane provide strong adhesive forces which prevent any seepage of liquefied n-octadecane.

**Table 2:** Surface area, porosity and latent heat of the SSPCM nanocomposites before and after the leakage study.

Sample	BET surface area (m^2^ g^−1^)	Pore volume (cm^3^ g^−1^)	Pore width (Å)	PCM loading (%)	Latent heat before (J g^−1^)		Latent heat after (J g^−1^)	
					∆Hm^a^ (J g^**−1**^)	∆Hc^b^ (J g^**−1**^)	∆Hm^c^ (J g^**−1**^)	∆Hc^d^ (J g^**−1**^)
SSPCM-C0	2	0.008	55	4.04	− 10.50	8.68	− 9.45	7.35
SSPCM-C5	3	0.006	58	4.93	− 12.81	9.76	− 10.54	8.31
SSPCM-C10	11	0.015	42	16.79	− 43.66	42.28	− 42.34	40.92
SSPCM-C30	12	0.012	49	22.14	− 57.56	55.10	− 56.22	54.90
SSPCM-C40	20	0.023	47	17.99	− 46.77	45.02	− 45.83	43.91

^a^Enthalpy on DSC melting curve before the leaching test, ^b^enthalpy on DSC freezing curve before the leaching test, ^c^enthalpy on DSC melting curve after the leaching test and ^d^enthalpy on DSC freezing curve after the leaching test.

The PCM loading shows that the SSPCM-C30 has the highest amount of PCM impregnated, with about 22% followed by SSPCM-C40 (18%), SSPCM-C10 (17%), SSPCM-C5 (5%) and SSPCM-C0 (4%). This indicates that SSPCM-C30 nanocomposite can hold more PCM compared to the other SSPCMs, due to its highly porous and high surface area of the C30 activated carbon which was used as the framework material. The BET surface area of the resulting SSPCM-C30 is low due to the high loading of n-octadecane, resulted in the high surface area reduction compared to C30. The loading percentage directly affects the latent heat value in which a higher amount of PCM loaded into the pores of AC will increase the ability of the nanocomposite to store the thermal energy.

**Figure 9:**
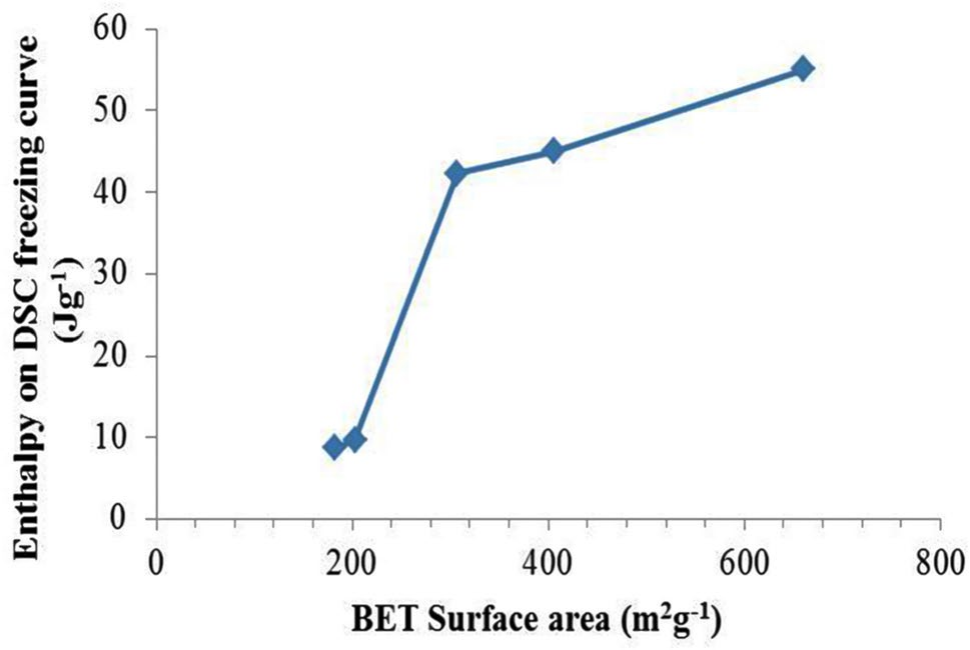
Plot of enthalpy derived from DSC freezing curve (J g^−1^) of the SSPCMs against BET surface area (m^2^ g^−1^) of the PKSACs used as the frameworks.

Figure 9 shows a plot of enthalpy on DSC freezing curve (J g^−1^) of SSPCM against BET surface area (m^2^ g^−1^) of their corresponding PKSACs. This indicates that the higher surface area of the PKSAC, the more PCM can be encapsulated into the pores and therefore more thermal energy can be stored.

**Figure 10:**
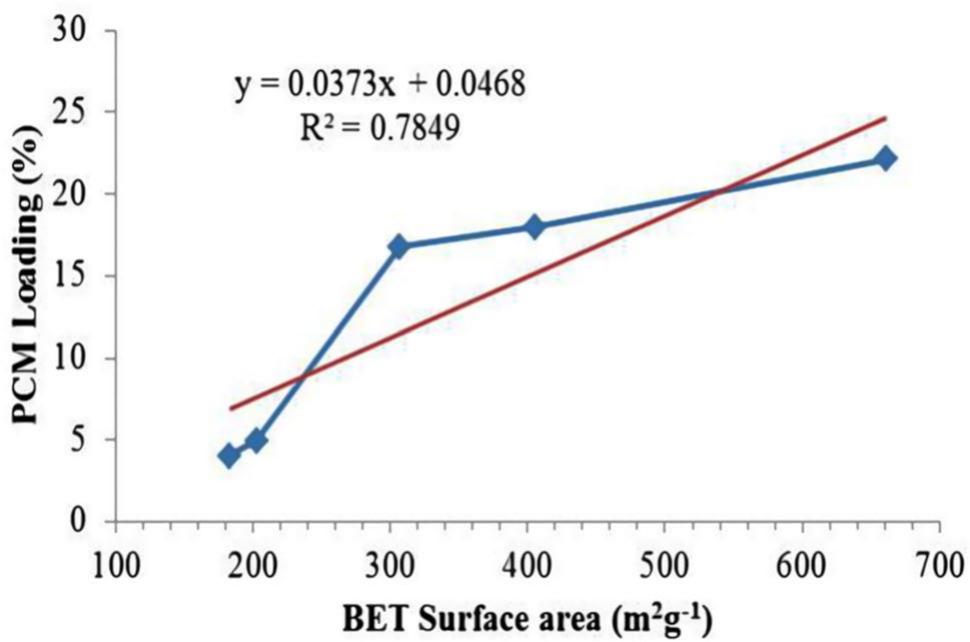
Plot of PCM loading (%) in the SSPCMs against BET surface area (m^2^ g^−1^) of the PKSACs used as the frameworks. The red straight line shows the linear fitting with the indicated correlation coefficient (R^2^).

Figure 10 shows the plot of PCM loading (%) of SSPCM against the BET surface area (m^2^ g^−1^) of their corresponding PKSACs. The straight-line plot shows the loading percentage of PCM is linearly related to surface area with high correlation coefficient of 0.78. High surface area of the PKSAC is directly affecting the PCM loading percentage, as more PCM can be stored and hold by the pores of the PKSACs with high surface area.

**Figure 11:**
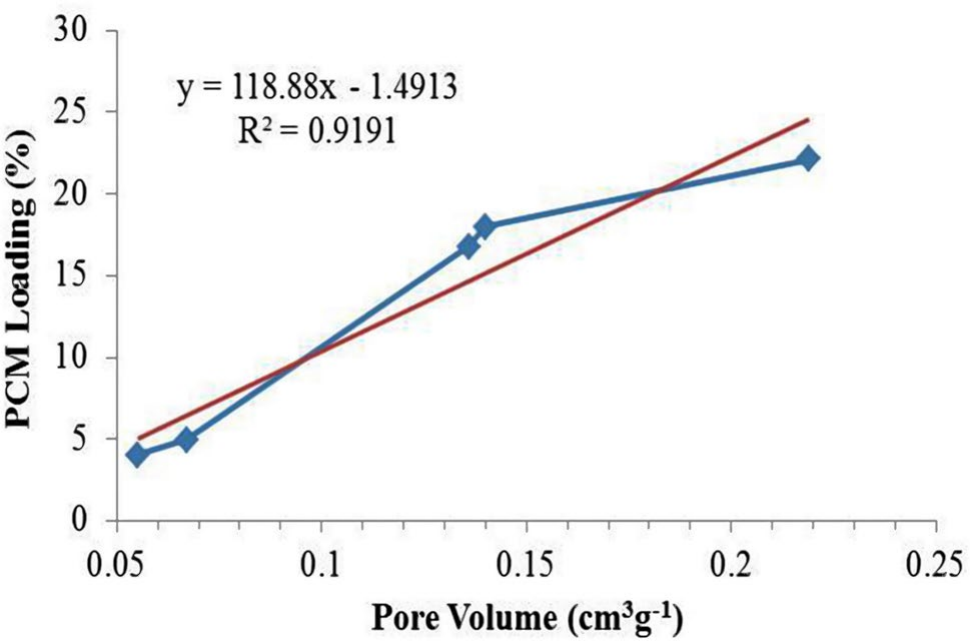
Plot of PCM loading (%) of SSPCM against pore volume (cm^3^ g^−1^) of the PKSACs used as the frameworks. The red straight line shows the linear fitting with the indicated correlation coefficient (R^2^).

Taking into account of very good correlation (0.91) between the PCM loading and pore volume observed in Fig. 11, this indicates that the loading percentage of PCM increase linearly with the pore volume. Besides that, the pore volume is linearly effect more PCM loading compared to BET surface area of the activated carbon. In general, this study shows that the surface area and pore volume of the PKSAC plays a crucial role in determining the amount of PCM to be encapsulated into the pores of PKSACs in the preparation of SSPCM, which subsequently determine the resulting TES properties.

### Leakage study

The objective of this test is to see the capability of the activated carbon to hold the n-octadecane in the temperature above the melting temperature of the n-octadecane. It was conducted by placing the SSPCM inside an oven at 80 ± 5 °C for 72 h (3 days). About 1 g of the SSPCM nanocomposite was weighed on a filter paper and exposed to 30 °C for 8 h. This is to study the ability of the activated carbon to hold the n-octadecane during the melting phase. The filter paper shows no leakage of n-octadecane, which indicates that the AC holds well the n-octadecane. Then, the samples were directly placed into an oven at 80 ± 5 °C for 3 days.

## Conclusion

A series of SSPCM nanocomposites prepared from activated carbons with different surface areas were successfully obtained. It was found that the higher the surface area and porosity of the activated carbon, the higher the PCM loading percentage, therefore the better the TES property of the resulting SSPCMs. The loading percentage of PCM was found to be linearly related to the surface area and pore volume of the AC, the host frameworks. This open up various applications, especially in building industry, textiles industry as well as microelectronics.

## Supplementary information

Supplementary Information 1.

## Data Availability

The data used to support the findings of this study are included in the article.
