# Supplementary material for: The effect of surface area on the properties of shape-stabilized phase change material prepared using palm kernel shell activated carbon
Source: Sci Rep. 2020 Sep 14;10:15047. doi: 10.1038/s41598-020-72019-1 (PMC7490345; doi:10.1038/s41598-020-72019-1)
Supplement: Supplementary file 1 — Supplementary Information 1. [file 41598_2020_72019_MOESM1_ESM.docx]

**THE EFFECT OF SURFACE AREA ON THE PROPERTIES OF SHAPE-STABILIZED PHASE CHANGE MATERIAL USING PALM KERNEL SHELL ACTIVATED CARBON AS THE FRAMEWORKS**

**Ahmad Fariz Nicholas^1^, Mohd Zobir Hussein^1,^*, Zulkarnain Zainal**^1^**, Tumirah Khadiran^2^,**

^1^Materials Synthesis and Characterization Laboratory, Institute of Advanced Technology (ITMA),

Universiti Putra Malaysia, 43400 Serdang Selangor, Malaysia

^2^Forest Product Division, Forest Research Institute Malaysia (FRIM), 52109 Kepong, Selangor, Malaysia

*Correspondence: [mzobir@upm.edu.my](mailto:mzobir@upm.edu.my); Tel.: +603-89468092

**Notes**: The supplementary data is the TGA/DTG thermograms of the ACs prepared before the impregnation of n-octadecane to become SSPCM. The comparison between the TGA/DTG thermograms of ACs and SSPCM proves that the impregnation of n-octadecane was successfully done.


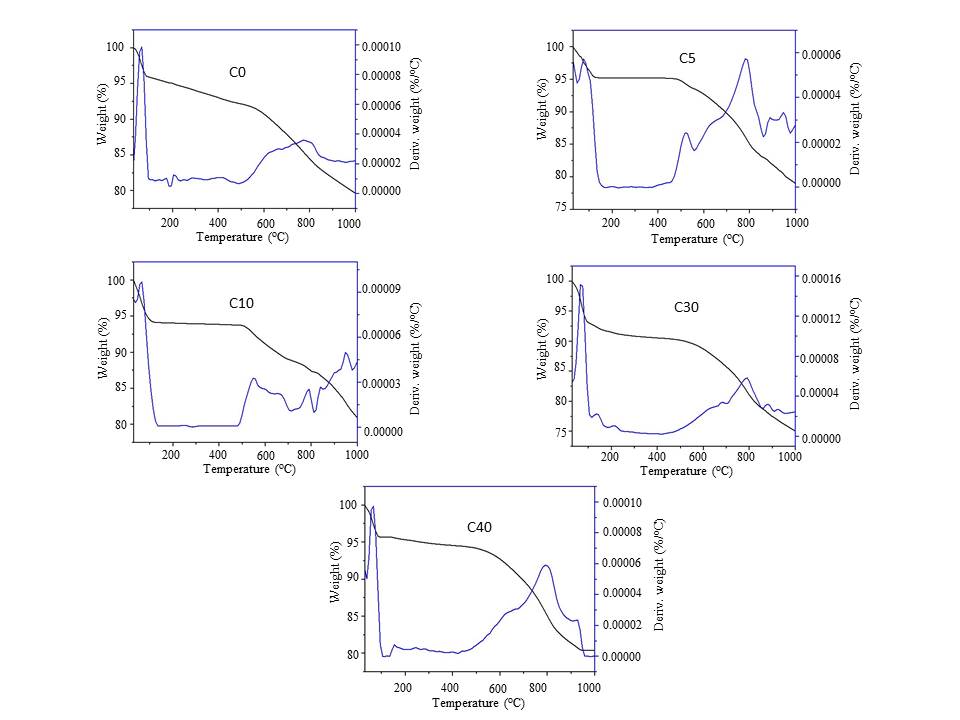
Supplementary Data: TGA/DTG thermograms of ACs (C0, C5, C10, C30 and C40)
